# Aerobic exercise training improves learning and memory performance in hypoxic-exposed rats by activating the hippocampal PKA–CREB–BDNF signaling pathway

**DOI:** 10.1186/s12868-025-00935-x

**Published:** 2025-02-21

**Authors:** Shichen Luo, Lei Shi, Tong Liu, Qiguan Jin

**Affiliations:** 1 College of Physical Education, Yangzhou University, Yangzhou, 225127 Jiangsu China; 2 Nanjing Yincheng Primary School, Nanjing Yincheng Primary School Education Group, Nanjing, 210036 Jiangsu China

**Keywords:** Altitude hypoxia, Aerobic exercise training, Learning and memory, PKA–CREB–BDNF, Synaptic plasticity, Hippocampus

## Abstract

**Background:**

This study aims to investigate the effects of aerobic exercise training on learning and memory (L&M) performance in rats exposed to altitude hypoxia and its relationship with hippocampal plasticity and the PKA–CREB–BDNF signaling pathway.

**Methods:**

Male Sprague–Dawley rats were exposed to 14.2% hypoxia with or without 60 min of non-weight-bearing swimming training for 8 weeks. The L&M performance was evaluated using the Morris water maze, and the mRNA expression of PSD95, SYP, PKA, CREB, CBP, and BDNF in the hippocampus was detected.

**Results:**

Chronic hypoxia exposure significantly impaired L&M performance and reduced the mRNA expression of hippocampal PSD95, SYP, PKA, CREB, CBP, and BDNF. Aerobic exercise training effectively reversed these changes by enhancing hippocampal synaptic plasticity through the activation of the PKA–CREB–BDNF signaling pathway.

**Conclusion:**

Aerobic exercise training can alleviate the decline in L&M performance caused by altitude hypoxia exposure, possibly through the activation of the hippocampal PKA–CREB–BDNF signaling pathway.

## Introduction

Learning and memory (L&M) is one of the most crucial high-level neural functions of the brain, and the maintenance of long-term memory requires the synthesis of new proteins, where the protein kinase A (PKA)-cAMP response element binding protein (CREB) signaling pathway plays an important role in the synthesis of new proteins. CREB is a protein found in the nucleus of eukaryotic cells and is present in various types of neurons in the brain, it may regulate gene transcription, playing a decisive role in the formation of L&M [1], and is recognized as an indispensable transcription factor for memory storage. The neurotransmitter promotes cyclic AMP (cAMP) synthesis in the postsynaptic membrane, activates PKA, and causes CREB phosphorylation. Phosphorylated CREB facilitates the transcription of the CRE sequence, subsequently regulating the expression of numerous downstream genes, including those encoding synaptophysin I and brain-derived neurotrophic factor (BDNF), affecting the survival and growth of neurons and synaptic plasticity. Thus promoting the formation of long-term memory [2, 3]. Different types of CREB dysfunction have impairments in memory and long-term potentiation (LTP) formation [4]. Therefore, hippocampal PKA-CREB-BDNF plays a very important role in L&M. However, the central nervous system (CNS) is most sensitive to hypoxia. Chronic altitude hypoxia can cause advanced brain dysfunction such as L&M, behavior, and emotion [5]. Acute hypoxia, chronic hypoxia, and intermittent hypoxia all lead to memory impairment and learning disabilities [6–8]. Glycolysis, oxidative stress, mitochondrial dysfunction, neurotoxicity, and inflammatory responses are the primary contributors to learning and memory deficits following acute hypoxia [9–11]. In contrast, chronic hypoxia-induced neurovascular coupling impairment, cell apoptosis, and transcription factor-mediated inflammation are the main contributors to functional deficits, and these are more challenging to recover from compared to acute injuries [12]. Proper exercise training can improve L&M performance [6, 7], but within different altitude hypoxic environments, what it may bring to L&M performance or its mechanism is still unclear. In this study, rats were subjected to 8 weeks of hypoxia exposure with or without aerobic exercise training. Learning and memory performance was assessed, and mRNA expression levels of postsynaptic density protein 95 (PSD-95), synaptophysin (SYP), PKA, CREB, CREB binding protein (CBP), and BDNF in the hippocampus were measured to explore the effects and interactions of hypoxia and exercise training on L&M and their relationship with hippocampal PKA-CREB-BDNF signaling pathway, to provide experimental basis for further research on the effects of exercise training on L&M performance and its mechanism in altitude hypoxic environment.

## Material and methods

### Animals and grouping

Forty 6-week-old male Sprague Dawley (SD) rats weighing 160–180 g were purchased from the Experimental Animal Center of Nantong University, license number SCXK (Su) 2019-0001. Place the rats in cages (485 * 350 * 200 mm), with 3–4 rats per cage. Sawdust is placed in the cage and replaced every 3 days. They were housed under the temperature of 20 ± 2 °C and with a 12-h light–dark cycle and allowed ad libitum access to food and water. The relative humidity is maintained at 60%–70%. After 3 days of adaptive feeding, rats were randomly divided into four groups: normoxic control group (NC), hypoxic control group (HC), normoxic exercise group (NE), and hypoxic exercise group (HE), with 9 rats in each group (1 animal in the HC group and 1 in the NE group died during the intervention, while 2 animals in the HE group drowned).

### Experimental treatment

NE and HE groups underwent no-weight-bearing swimming training in the afternoon, six times a week for 8 weeks. Adaptive training was conducted during the first week, with the exercise duration gradually increasing to 60 min by the end of the week [8]. The swimming pool was a rectangular plastic container (120 cm × 80 cm × 70 cm), with smooth inner walls, a water depth of over 50 cm, and a 30–33 °C water temperature. The rats’ condition was monitored during swimming to prevent accidents such as drowning, and promptly clean the pool by removing any feces to maintain water hygiene. The hypoxic groups (HC, HE) were housed and trained in a hypoxic chamber (The MAG-10 Mountain Air Generator, Higher Peak Company, USA). During the first 2 weeks, the simulated altitude in the chamber was gradually increased from 1600 to 3000 m, with a final oxygen concentration of 14.2%. A Morris water maze (MWM) test was performed in the 8th week of the experiment to assess L&M performance[9]. The experimental procedures were approved by the Experimental Animal Management Committee and Experimental Animal Ethics Committee of Yangzhou University (YZUDWWL-202107003).

### Sample collection

After completing the MWM test, the rats were fasted for 12 h and anesthetized via intraperitoneal injection of 2% pentobarbital sodium solution at the dose of 50 mg/kg. Following careful dissection, the whole brain was removed, and the hippocampus was separated on ice. A 1 × 1 × 1 mm block was cut from the CA1 region of the right hippocampus of the treated rats and fixed in 2.5% glutaraldehyde solution. The remaining hippocampal tissue was immediately placed in liquid nitrogen in cryogenic tubes and then transferred to − 80 °C for storage. Total RNA was extracted and determine the expression of PKA, CREB, CBP, and BDNF mRNA in the hippocampal tissues.

### Index testing

#### MWM test

In the MWM test, animals must locate the submerged escape platform from a starting position in the open swimming pool, using perimeter cues. The experimental apparatus was placed in a separate laboratory to avoid external interference, with visual cues consisting of plastic plates of various colors and shapes hanging on the shelf. Four light sources were positioned outside the curtain to enhance visibility. The water was made opaque with white dye to allow tracking of the mice. The maze consisted of a circular pool with a diameter of 120 cm and a height of 45 cm, filled with water maintained at 21 ± 1 °C. The circular pool was divided into 4 equal quadrants and established 4 starting positions in each quadrant. The escape platform was a 10 cm diameter plexiglass disk located in the third quadrant, submerged 1 cm beneath the water surface. For each trial, the animals were placed into the water facing the wall, and their swimming trajectory from the starting position to the escape platform was recorded using the Animal Video Analysis System V2.0 (Anhui Zhenghua, China).

In the 8th week of the experiment, positioning navigation training was conducted in the MWM from 8:00 a.m. to 11:00 a.m. for 5 consecutive days. Each rat underwent 3 trials per day with a 60-s interval between trials. The training began in the first, second, and fourth quadrants quadrants in turn, and the rats facing the pool wall into the water, and the route map and time (escape latency) required to find and climb the platform within 120 s were recorded, and 10 s were allowed to stay on the platform to strengthen the memory effect. If the rat does not find the platform within the prescribed test time of 120 s, it must be guided to the platform, and it is also allowed to stay on the platform for 10 s. On the morning of the sixth day, a positioning navigation test was conducted, and the average value of three incubation periods was taken as the escape latency. In the afternoon, a spatial exploration test was performed, that is, the platform was removed, and the rats were placed into the water facing the pool wall from the same water entry point. The trajectory of the rats' swimming over the 120 s and the times they crossed the corresponding position of the platform were measured.

### Transmission electron microscopy (TEM)

Hippocampal tissue was extracted from the glutaraldehyde solution, routinely embedded, and ultra-thin sections were prepared and stained with lead. Changes in synaptic and mitochondrial structures were observed using a Hitachi HT7800 transmission electron microscope. At least three fields per slice were examined under low magnification (10,000×) to evaluate the number of synapses. Additionally, at least five fields per slice were observed under high magnification (25,000×) to measure the thickness and length of the postsynaptic density (PSD) using ImageJ 10. Three independent experiments were performed in each group.

### RT-PCR

mRNA expression levels of PSD-95, SYP, PKA, CREB, CBP, and BDNF were determined by RT-PCR. Total RNA was extracted from the hippocampus using the Trizol method (Life Technologies). RNA purity was assessed by measuring the 260/280 absorbance ratio with a NanoDrop ND-3300 microspectrophotometer (a ratio between 1.8 and 2.0 was considered acceptable for pure RNA). cDNA synthesis was carried out following the instructions of the cDNA synthesis kit (TOYOBO (Shanghai) Biotechnology Co., LTD) using a 2720 Thermal Cycler gradient PCR instrument (USA).. After the reaction, the cDNA was stored at − 20 °C. The gene was then amplified using the SYBR Green Master (ROX) kit (Roche Diagnostics) with cDNA as a template and GAPDH as the internal reference, employing an ABI 7500 RT-PCR system. The amplification protocol was as follows: pretreatment at 50 °C for 2 min, predenaturation at 95 °C for 10 min, denaturation at 95 °C for 15 s, and annealing at 60 °C for 60 s, with a total of 40 cycles. After amplification, the Ct value of the target gene was normalized to GAPDH, and the relative expression of the target gene was calculated [11]. Primers for PSD-95, SYP, PKA, CREB, CBP, BDNF, and GAPDH were designed and synthesized by Shanghai BioEngineering Co., LTD. The primer sequence is shown in Table 1.

**Table 1:** Primer sequence used in the study

Gene Name	Primers	Sequence (5ʹ to 3ʹ)
PSD95	Forward	AGACCGACGACATTGGCTTCATTC
	Reverse	TCCATCTGGGTCACCGTCTCATAG
SYP	Forward	GTGCCAACAAGACGGAGAGT
	Reverse	AGTAGTCCCCAACCAGGAAGAT
PKA	Forward	TTGACGACTATGAGGAGGAAGA
	Reverse	CACAAGCACACCCCTAAAACT
CREB	Forward	CAGAGTGGAGATGCTGCTGTAA
	Reverse	ATGGATACCTGGGCTAATGTG
CBP	Forward	CAAGATGGGGATGACTGGTAAC
	Reverse	GCTGGCTAACTGAGGGTTCACT
BDNF	Forward	GATGCCAGTTGCTTTGTCTTC
	Reverse	TAAAATCTCGTCTCCCCAACA
GAPDH	Forward	ACAGCAACAGGGTGGTGGAC
	Reverse	TTTGAGGGTGCAGCGAACTT

### Statistical analysis

The experimental data were processed using SPSS 22.0 statistical software, and the results are presented as mean ± standard deviation (M ± SD). The main effect and interaction effect of hypoxia and exercise were analyzed by two-way ANOVA, and the group mean differences was tested by Tukey’s HSD test. *P* ≤ 0.05 was considered statistically significant, and *P* ≤ 0.01 was considered highly significant.

## Results

### Changes in learning and memory ability in each group of rats

**Fig. 1:**
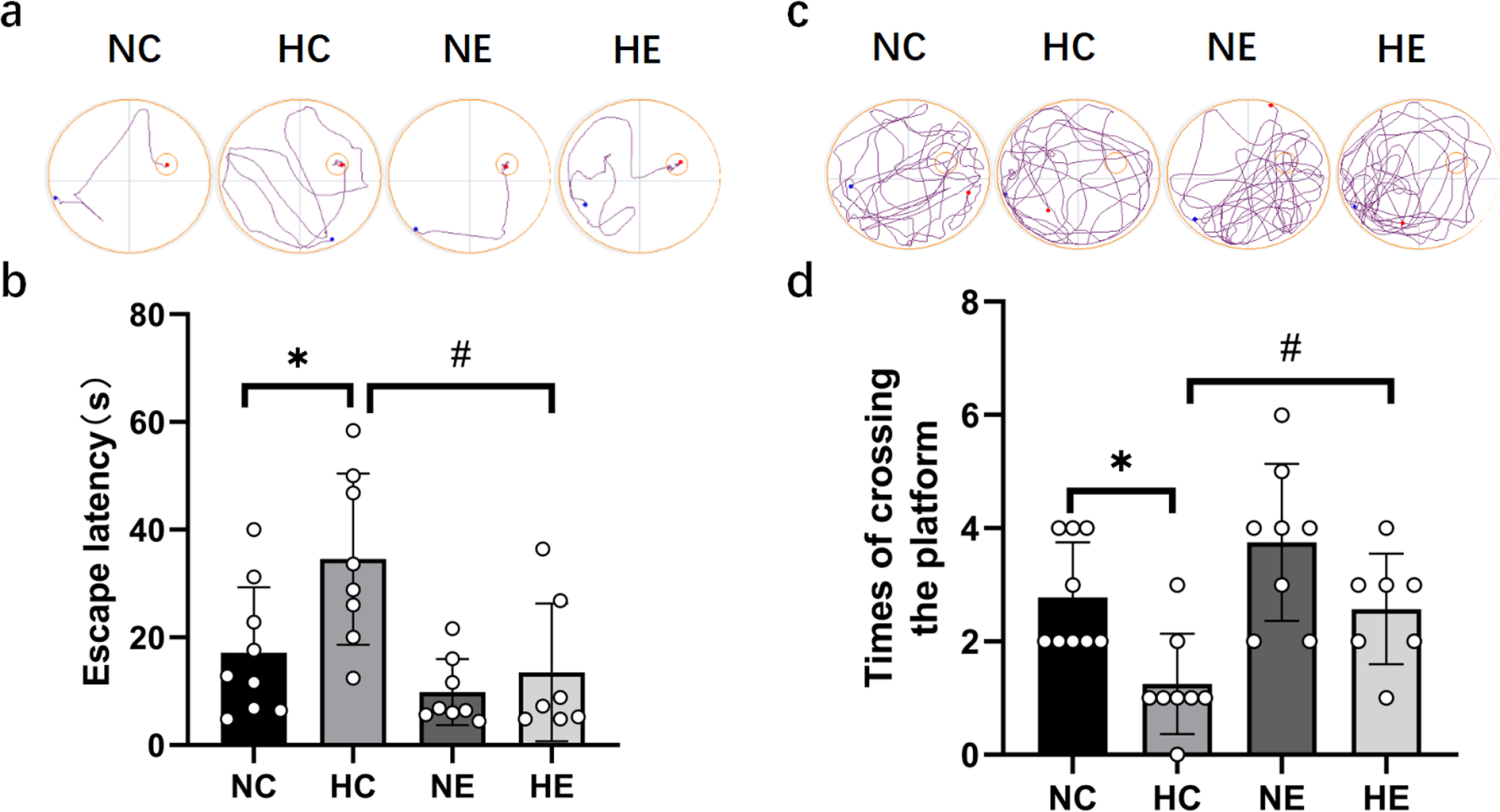
Results of the MWM test. *NC* Normoxic control group, *NE* Normoxic exercise group, *HC* hypoxic control group, *HE* Hypoxic exercise group. vs. NC: * *P* < 0.05, ** *P* < 0.01; vs HC: ## *P* < 0.01

According to the two-way ANOVA of NC, HC, NE and HE groups, hypoxia combined with exercise training had no significant interaction effect on shortening the escape latency or increasing the number of platform crossings. However, chronic hypoxia exposure significantly prolonged the escape latency and significantly reduced the number of platform crossings. Exercise training significantly reduced the escape latency and significantly increased the number of platform crossings, as shown in Fig. 1. The escape latency in the HC group was dramatically extended compared with the NC group, and the times of crossing the platform was significantly reduced. In contrast, the escape latency in the NE group was shortened, and the number of platform crossings was increased, although no significant difference was found. Compared to HC group, the escapes latency in HE group was markedly shortened, and the number of platform crossings was significantly increased. The data from the first five training sessions are presented in Fig. 1e.

### Changes in mRNA expression of PSD-95 and SYP in the hippocampus of rats in each group

**Fig. 2:**
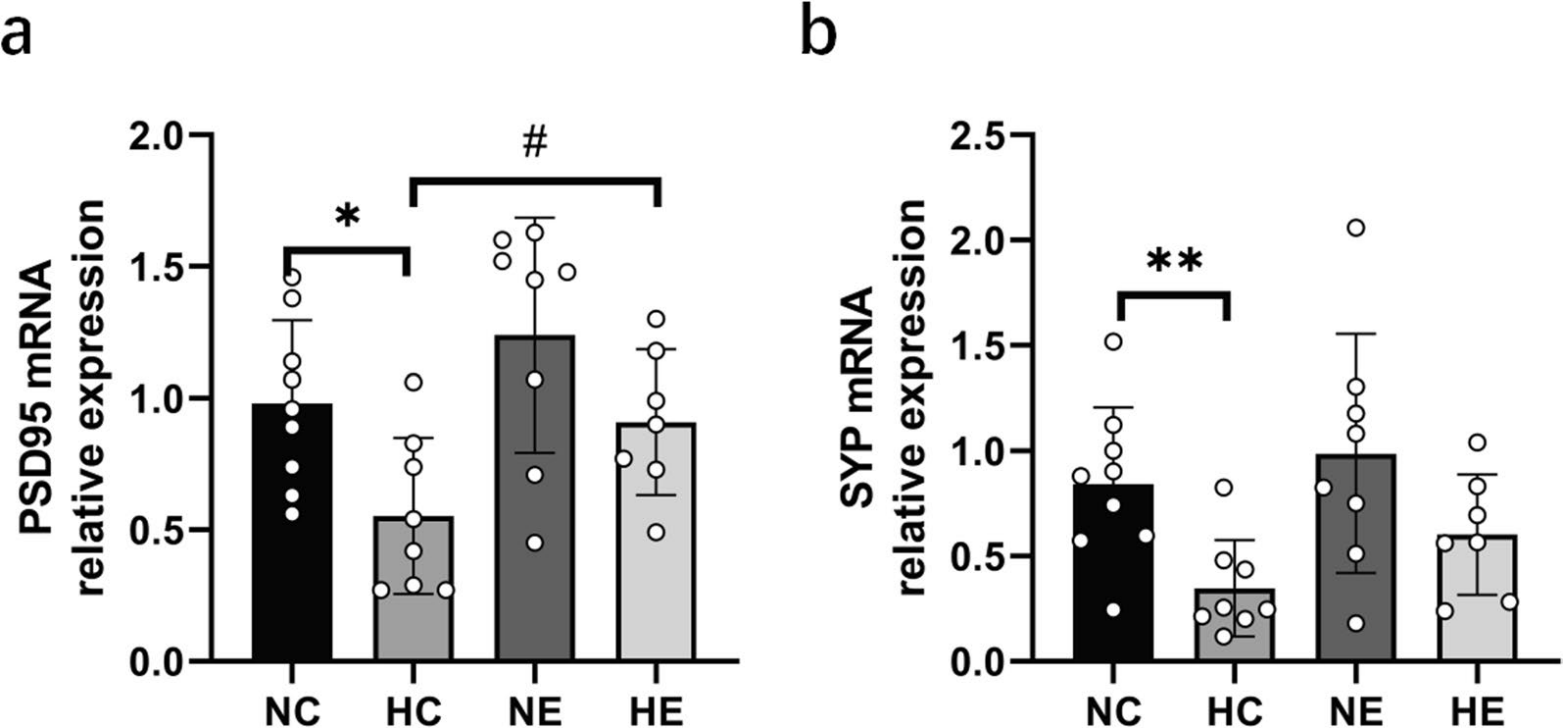
The expression changes of hippocampal PSD-95 and SYP mRNA in each group. *NC* Normoxic control group, *NE* Normoxic exercise group, *HC* hypoxic control group, *HE* Hypoxic exercise group. vs. NC: * *P* < 0.05, ** *P* < 0.01; vs HC: # *P* < 0.05

As shown in Fig. 2, two-way ANOVA of the NC, HC, NE, and HE groups revealed that hypoxia and exercise training had no significant interaction effect on improving the mRNA expression of PSD-95 or SYP in the hippocampus. Hypoxia exposure significantly decreased the mRNA expression of PSD-95 and SYP in the hippocampus. Long-term exercise training significantly increased the mRNA expression of PSD-95 in the hippocampus and also increased the mRNA expression of SYP, although no significant difference was observed. Compared to the NC group, the mRNA expressions of PSD-95 and SYP in the hippocampus of the HC group were markedly decreased. In the NE group, the mRNA expressions of PSD-95 and SYP in the hippocampus were increased, but no significant difference was found. Compared to the HC group, the mRNA expression of PSD-95 in the hippocampus of the HE group was significantly increased, while the mRNA expression of SYP was increased, though no significant difference was observed.

### Changes in hippocampal ultrastructure in each group

**Fig. 3:**
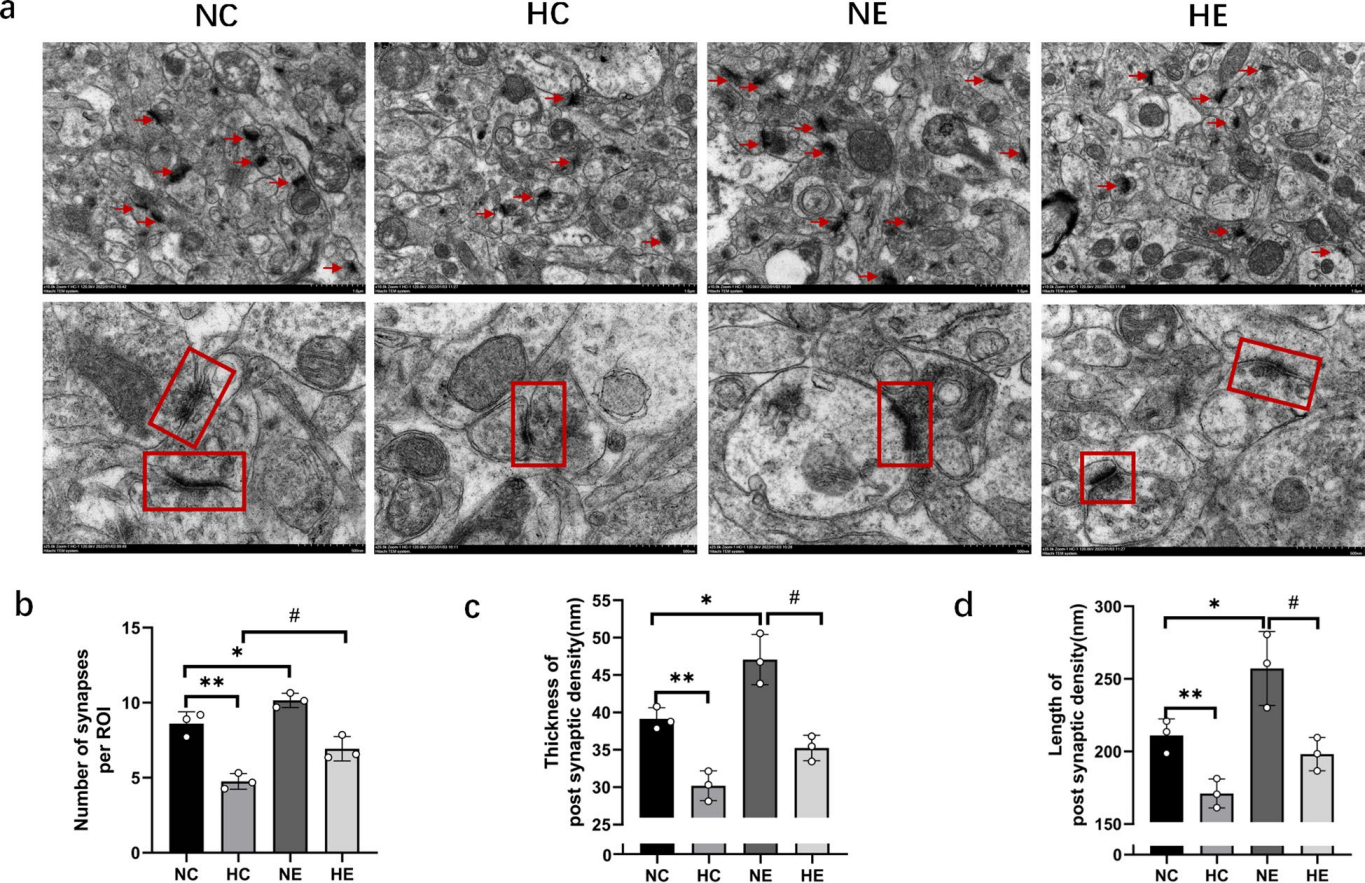
Changes of hippocampal ultrastructure in each group. **a** Electron microscopic observation of synaptic structure in CA1 region of hippocampus, red arrows and red boxes: synaptic structure, with scales of 1 µm (10,000×) and 200 nm (25,000×); **b**–**d** quantitative statistical results of the number of synapses, the thickness and the length of PSD, respectively. The data are expressed as Mean ± SD, N = 3. *NC* Normoxic control group, *NE* Normoxic exercise group, *HC* hypoxic control group, *HE* Hypoxic exercise group. vs. NC: * *P* < 0.05, ** *P* < 0.01; vs HC: #*P* < 0.05

Figure 3a shows representative TEM images of synapses of hippocampus neurons in all groups, In the NC group, the hippocampus exhibited a clear synaptic gap, thick postsynaptic density (PSD), and abundant synaptic vesicles with well-defined structures gathered in the presynaptic membrane. Mitochondria had normal structure with visible, well-organized cristae. In the HC group, hippocampal synapses were reduced, with a blurred synaptic gap and smaller synaptic interfaces. The mitochondrial structure was deformed and fuzzy. The group NE exhibited a large number of hippocampal synapses, a clear synaptic gap, and large synaptic interfaces with pronounced curvature. The thickness of the PSD was significantly increased, with abundant synaptic vesicles gathered in the presynaptic membrane, and the number of mitochondria also increased with clear structure. Compared with the HC group, the HE group showed increases in all parameters, including the number of hippocampal synapses, synaptic vesicles, PSD thickness, and the number of mitochondria, with nearly intact mitochondrial structure. As shown in Fig. 3b–d two-way ANOVA of the NC, HC, NE, and HE groups revealed that hypoxia and exercise training had no significant interaction effects on the number of synapses, PSD thickness, or PSD length in the hippocampus. chronic hypoxia exposure dramatically decreased the number of hippocampal synapses, PSD thickness, and PSD length, while exercise training significantly increased these parameters. Compared to the NC group, the number of hippocampal synapses, PSD thickness, and length in the HC group were significantly decreased. In the NE group, the number of hippocampal synapses, PSD thickness, and length were significantly increased. Compared to the HC group, the number of hippocampal synapses, PSD thickness, and length in the HE group were markedly increased.

### Changes in hippocampal BDNF mRNA expression in each group of rats

**Fig. 4:**
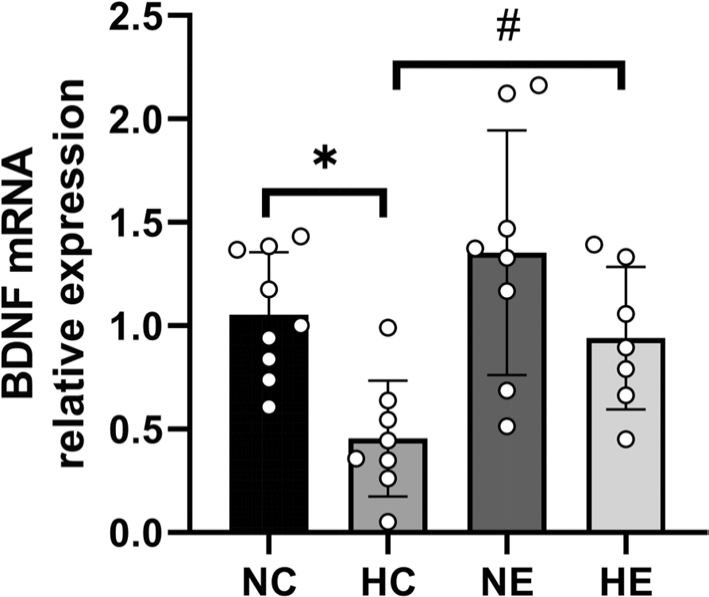
Changes of hippocampal BDNF mRNA expression in each group. *NC* Normoxic control group, *NE* Normoxic exercise group, *HC* hypoxic control group, *HE* Hypoxic exercise group. vs. NC: * *P* < 0.05; vs HC: # *P* < 0.05

As shown in Fig. 4, two-way ANOVA of the NC, HC, NE, and HE groups revealed that hypoxia and exercise training had no significant interaction effect on increasing the expression of BDNF mRNA in the hippocampus.Chronic hypoxia exposure dramatically decreased the expression of BDNF mRNA in the hippocampus, while exercise training significantly increased BDNF mRNA expression. Compared with the NC group, the hippocampal BDNF mRNA expression in the HC group was markedly decreased, while the hippocampal BDNF mRNA expression in the NE group was increased, but there was no significant difference. Compared to the HC group, hippocampal BDNF mRNA expression in the HE group was dramatically increased.

### Changes of mRNA expression of hippocampal PKA, CREB and CBP in each group

**Fig. 5:**
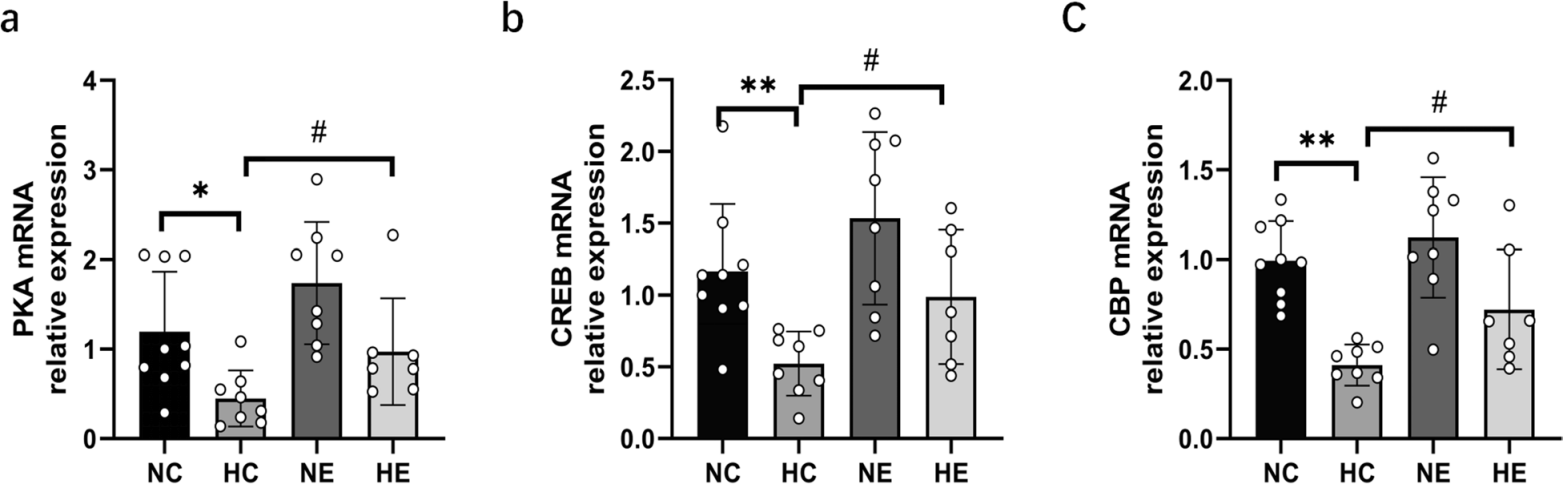
Changes of mRNA expression of hippocampal PKA, CREB and CBP in each group. *NC* Normoxic control group, *NE* Normoxic exercise group, *HC* hypoxic control group, *HE* Hypoxic exercise group. vs. NC: * *P* < 0.05, ** *P* < 0.01; vs HC: # *P* < 0.05

As shown in Fig. 5, two-way ANOVA of the NC, HC, NE, and HE groups revealed that hypoxia and exercise training had no significant interaction effect on the expression of PKA, CREB, and CBP mRNA in the hippocampus, chronic hypoxia exposure can dramatically down-regulate the mRNA expression of PKA, CREB and CBP in the hippocampus, while exercise training significantly up-regulated the mRNA expression of PKA, CREB, and CBP. Compared to the NC group, the mRNA expressions of hippocampal PKA, CREB, and CBP in HC group were significantly decreased. In the NE group, the mRNA expressions of PKA, CREB, and CBP in the hippocampus were increased, although no significant difference was observed.

## Discussion

Research indicates that the impact of hypoxia exposure on L&M performance is closely associated with the mode, duration, and severity of hypoxia exposure. Cognitive function initially declines, then improves, and ultimately deteriorates with prolonged altitude exposure [12]. As the altitude rises, the adaptation time to the plateau also extends correspondingly. Higher altitudes exacerbate the physiological effects of hypoxia, leading to more pronounced declines in work capacity [13]. As the time of high-altitude environmental exposure increased, the attention, information processing rate, spatial cognitive ability, and executive function of the subjects all revealed different degrees of impairment. The longer the exposure time, the damage to cognitive function is more serious. Therefore, chronic altitude hypoxia will lead to degenerative changes in brain tissue, resulting in reduced cognitive function and L&M performance [14–16]. Regular and appropriate aerobic exercise has been shown to enhance cerebral vasculature and neural activity, thereby improving cognitive functions [17–21]; an inverted U-shaped dose–response relationship exists between exercise intensity and memory performance, with lower intensity enhancing short-term delayed working memory and moderate to higher intensity improving long-term delayed working memory [21]. Despite extensive research on the effects of hypoxia and exercise training on L&M performance, studies specifically examining exercise training under altitude hypoxia conditions remain limited. A recent review suggests that various exercise characteristics may influence the relationship between exercise and cognitive performance under hypoxia. The review found that hypoxia exposure impairs some, but not all, aspects of cognitive function. Exercising under hypoxia can have small to moderate positive effects on certain aspects of cognitive function. Under hypoxia, the exercise-cognition relationship is moderated by factors such as age, cognitive task type, exercise modality, intensity, training type, and hypoxia level. Moderate-intensity exercise under hypoxia may enhance cerebral blood flow and offset reductions in SpO2, thereby improving cognitive function. Notably, exercising under moderate hypoxia provides greater cognitive benefits compared to severe hypoxia. Chronic exercise exerts more significant cognitive benefits than acute exercise under hypoxia. Furthermore, moderate-intensity exercise under hypoxia has been shown to enhance cognitive function [22]. To further investigate the effects and interaction of altitude hypoxia and aerobic exercise on L&M performance, this study employed an interactive experimental design. Rats were exposed to 14.2% hypoxia for 8 weeks and/or engaged in 60 min of non-weight-bearing swimming training. L&M performance was assessed using the MWM. The results indicated that altitude hypoxia and exercise training did not have a significant interaction effect on reducing escape latency or increasing platform crossings in rats. Long-term altitude hypoxia could significantly prolong the escape latency and decrease the times of crossing the platform, while aerobic exercise training could not significantly shorten the escape latency and decrease the times of crossing the platform under normoxia. However, aerobic exercise training under altitude hypoxia significantly reduced escape latency and increased platform crossings. It further proves that long-term altitude hypoxia exposure can inhibit L&M performance, and appropriate exercise training can significantly reduce the inhibition of L&M performance induced by altitude hypoxic exposure. This is consistent with the findings of Koester et al., who suggested that exercise is profitable for recovering the cognitive function impaired by High-altitude exposure [23]. Thus, individuals living in high-altitude hypoxic environments can mitigate cognitive impairment caused by hypoxia through regular exercise, which may play a crucial role in enhancing cognitive function.

Synaptic plasticity refers to the capacity of synapses to adjust their function, morphology, and number under specific conditions, encompassing alterations in synaptic structure and transmission efficiency [24]. Neuronal plasticity enables the CNS to acquire new skills, consolidate and retrieve memories, reorganize neural networks in response to stimuli, and recover from damage. Neuronal plasticity may occur using neurogenesis, cellular apoptosis, synaptic-dependent activity, and the reorganization of neuronal networks. The hippocampus, a delicate structure situated in the medial temporal lobe, serves as a critical hub for L&M. This region is highly susceptible to ischemia, hypoxia, inflammation, and epilepsy. Research has shown that chronic intermittent hypoxia significantly reduces hippocampal spine density and PSD thickness [25]. Exercise rehabilitation training significantly increases the number of synapses and mitochondria in the dentate gyrus of ischemic rats, promoting changes in synaptic structural plasticity [26]. This study examined hippocampal ultrastructural changes to investigate the physiological mechanisms by which altitude hypoxia and exercise training influence L&M. Compared to the NC group, the HC group exhibited a reduced number of synapses in the hippocampal CA1 region, smaller synaptic interfaces, deformed synaptic gaps, and blurred mitochondrial structures. In the NE group, synapse numbers increased, synaptic interface curvature was enhanced, synaptic gaps were clear, PSD thickness was significantly greater, and presynaptic vesicle numbers were elevated. Mitochondrial numbers increased, with more regular shapes and uniform sizes. Compared to the HC group, the HE group exhibited increased numbers of synapses and synaptic vesicles in the hippocampus, greater PSD thickness, a higher number of mitochondria, clearer boundaries, and reduced mitochondrial folding. Quantitative measurements revealed that hippocampal synapse numbers, PSD thickness, and PSD length were significantly reduced in the HC group but notably increased in the NE group compared to the NC group. Compared to the HC group, the HE group showed a remarkable increase in hippocampal synapse numbers, PSD thickness, and PSD length. These results further indicate that chronic hypoxia exposure can reduce the plasticity of hippocampal synaptic structure, while appropriate exercise training can significantly increase the plasticity of hippocampal CA1 synaptic structure under normoxia and hypoxia conditions, which has a certain protective effect on the damage of hippocampal synaptic structure in rats exposed to hypoxia. Research indicates that SYP and PSD95 are critical for synaptic function, playing key roles in synaptic plasticity, stable synaptic changes, and serving as “bridges” in synaptic connections [27]. Reduced expression levels of SYP and PSD95 proteins indicate degenerative changes in pre- and post-synaptic structures, reduced synapse numbers, impaired synaptic vesicle transport capacity, and disrupted glutamatergic post-synaptic signal transduction and integration. These deficits hinder neural transmission, information processing, and storage, ultimately affecting LTP and LTD formation. As a result, cognitive functions, including L&M, are compromised. Chronic intermittent hypoxia significantly reduces PSD95 and SYP expression in the hippocampus of rats [28]. Long-term exercise training not only increases SYP and PSD-95 expression in the ischemic brain, promoting neural function recovery [29, 30], but also enhances their expression in the hippocampus of rats with chronic stress and type 2 diabetes, thereby improving spatial learning and memory [31, 32]. However, the combined effects of altitude hypoxia and exercise training on PSD-95 and SYP expression in the hippocampus remain unexplored. This study assessed hippocampal mRNA expression levels of PSD-95 and SYP in rats following 8 weeks of 14.2% hypoxia exposure and/or 60 min of non-weight-bearing swimming, using an interactive experimental design. The results showed that there was no significant interaction between altitude hypoxia and aerobic exercise training in enhancing the expression of PSD-95 and SYP mRNA in the hippocampus. Long-term altitude hypoxia could significantly down-regulate the expression of PSD-95 and SYP mRNA in the hippocampus, while aerobic exercise could not significantly up-regulate the expression of PSD-95 and SYP mRNA in the hippocampus under normal oxygen. However, it can significantly up-regulate the expression of PSD-95mRNA in the hippocampus under altitude hypoxia. In conclusion, the mRNA expression changes of PSD-95 and SYP in the hippocampus during chronic hypoxia exposure or aerobic exercise training are consistent with the changes in synaptic ultrastructure and learning and memory ability. These findings suggest that chronic hypoxia exposure and exercise training modulate hippocampal synaptic plasticity by regulating PSD-95 and SYP mRNA expression, potentially impacting L&M performance.

BDNF, a neurotrophic factor widely expressed in the central nervous system, is crucial for hippocampal structural and functional integration, playing a vital role in brain development, maintenance, and neuronal plasticity. Research shows that 24 h of acute hypoxia exposure significantly reduces spatial L&M and hippocampal BDNF levels in rats, which recover after 72 h of hypoxia exposure [33]. Seven days of hypoxia exposure significantly downregulates BDNF protein expression in the hippocampus of rats [34]. Long-term aerobic exercise training promotes hippocampal BDNF synthesis and release, facilitating neuronal growth, synaptic plasticity, and enhanced L&M performance [35–38]. Conversely, other studies suggest that moderate hypoxia combined with exercise enhances synaptic plasticity through increased BDNF expression. The hypoxia- and exercise-induced upregulation of BDNF can facilitate cerebral neural activation and neurogenesis and, therefore, lead to cognition improvements [39, 40]. This study investigated the effects and interactions of hypoxia and exercise training on hippocampal BDNF expression by assessing BDNF mRNA levels following 8 weeks of 14.2% hypoxia exposure and/or 60 min of weightless swimming training.. The results revealed no significant interaction between hypoxia and exercise training in improving hippocampal BDNF mRNA expression. Long-term altitude hypoxia significantly reduced hippocampal BDNF mRNA levels. Although aerobic exercise training did not significantly upregulate hippocampal BDNF mRNA expression under normoxia, it effectively reversed hypoxia-induced reductions in hippocampal BDNF mRNA levels. Thus, under altitude hypoxia, appropriate aerobic exercise training improves hippocampal BDNF mRNA expression, thereby enhancing L&M performance.

However, the expression of BDNF is regulated by PKA-CREB [1]. Sakamoto et al. [41] believe that CREB is a key factor in physiological processes such as neural development, neuron survival, and synaptic plasticity, and is closely related to the L&M mechanism of the brain. The phosphorylation level of CREB is positively correlated with the activation of PKA, and the transcriptional activity of the CREB-centered transcription complex needs to be increased with cofactor CBP in a stimulus-dependent manner, to increase the activity of CRE promoter to activate downstream genes. Thus, synaptic plasticity in the hippocampus can be regulated through the PKA-CREB-BDNF signaling pathway [42, 43]. In the PKA-CREB-BDNF signaling pathway, a variety of neurotransmitters or neuropeptides can bind to G protein-coupled receptors (GPCR), resulting in increased cAMP cyclase activity, and activation of PKA through increased cAMP level to phosphorylate CREB Ser-133. This enables CREB to bind to CBP and activate the transcription of its target gene BDNF mRNA [44]. Activating CREB and its upstream signaling pathway through targeted drugs can improve the learning and memory function of patients with learning and memory dysfunction or healthy people [45]. However, hypoxia could downregulate the expression of BDNF via increasing the phosphorylation of ERK and phosphorylation of CREB at Ser142 reducing the phosphorylation of CREB at Ser133 [28, 46] and significantly reducing the expression of the PSD-95 in the hippocampus [25].

Moderate-intensity exercise training enhances hippocampal plasticity through the Akt-CREB-BDNF signaling pathway, improving spatial L&M performance [47, 48]. The combined effects of hypoxia and exercise training on hippocampal PKA, CREB, and CBP expression remain unclear. This study examined the mRNA expression of PKA, CREB, and CBP in the hippocampus of rats following 8 weeks of 14.2% hypoxia exposure with/without 60 min of non-weight-bearing swimming using an interactive experimental design. The results showed that hypoxia combined with exercise training had no significant interaction on up-regulating the mRNA expression of PKA, CREB, and CBP in the hippocampus, while long-term altitude hypoxia could significantly down-regulate the mRNA expression of PKA, CREB, and CBP in the hippocampus. Although exercise training could not significantly up-regulate the mRNA expression of PKA, CREB, and CBP in the hippocampus under normoxia, it could significantly reverse the expression of PKA, CREB, and CBP mRNA in the hippocampus under altitude hypoxia. In conclusion, the mRNA expression changes of PKA, CREB, and BDNF in the hippocampus of rats during chronic hypoxia exposure or exercise training are consistent with the changes in L&M performance, indicating that long-term altitude hypoxia exposure inhibits hippocampal PKA–CREB–BDNF signaling pathway, which may be the pathophysiological mechanism of L&M impairment induced by hypoxia. Conversely, appropriate aerobic exercise training under hypoxic conditions at high altitudes effectively mitigates L&M impairment by up-regulating the hippocampal PKA–CREB–BDNF signaling pathway.

## Conclusion

In summary, long-term altitude hypoxia exposure impairs L&M performance by suppressing the hippocampal PKA-CREB-BDNF signaling pathway, whereas aerobic exercise training significantly reverses altitude hypoxia-induced declines in L&M performance. Chronic hypoxia exposure and exercise training modulate hippocampal PKA-CREB-BDNF expression, which may serve as a key mechanism influencing L&M performance.

## Data Availability

Data is provided within the manuscript or supplementary information files.
